# Artificial Intelligence–Powered Training Database for Clinical Thinking: App Development Study

**DOI:** 10.2196/58426

**Published:** 2025-01-03

**Authors:** Heng Wang, Danni Zheng, Mengying Wang, Hong Ji, Jiangli Han, Yan Wang, Ning Shen, Jie Qiao

**Affiliations:** 1 Education Department, Peking University Third Hospital, Beijing, China; 2 Information Management and Big Data Center, Peking University Third Hospital, Beijing, China; 3 Department of Cardiology, Peking University Third Hospital, Beijing, China; 4 Department of Obstetrics and Gynecology, Peking University Third Hospital, Beijing, China; 5 Department of Pulmonary and Critical Care Medicine, Peking University Third Hospital, Beijing, China; 6 Centre for Reproductive Medicine, Department of Obstetrics and Gynecology, Peking University Third Hospital, Beijing, China

**Keywords:** artificial intelligence, clinical thinking ability, virtual medical records, distance education, medical education, online learning

## Abstract

**Background:**

With the development of artificial intelligence (AI), medicine has entered the era of *intelligent medicine*, and various aspects, such as medical education and talent cultivation, are also being redefined. The cultivation of clinical thinking abilities poses a formidable challenge even for seasoned clinical educators, as offline training modalities often fall short in bridging the divide between current practice and the desired ideal. Consequently, there arises an imperative need for the expeditious development of a web-based database, tailored to empower physicians in their quest to learn and hone their clinical reasoning skills.

**Objective:**

This study aimed to introduce an app named “XueYiKu,” which includes consultations, physical examinations, auxiliary examinations, and diagnosis, incorporating AI and actual complete hospital medical records to build an online-learning platform using human-computer interaction.

**Methods:**

The “XueYiKu” app was designed as a contactless, self-service, trial-and-error system application based on actual complete hospital medical records and natural language processing technology to comprehensively assess the “clinical competence” of residents at different stages. Case extraction was performed at a hospital’s case data center, and the best-matching cases were differentiated through natural language processing, word segmentation, synonym conversion, and sorting. More than 400 teaching cases covering 65 kinds of diseases were released for students to learn, and the subjects covered internal medicine, surgery, gynecology and obstetrics, and pediatrics. The difficulty of learning cases was divided into four levels in ascending order. Moreover, the learning and teaching effects were evaluated using 6 dimensions covering systematicness, agility, logic, knowledge expansion, multidimensional evaluation indicators, and preciseness.

**Results:**

From the app’s first launch on the Android platform in May 2019 to the last version updated in May 2023, the total number of teacher and student users was 6209 and 1180, respectively. The top 3 subjects most frequently learned were respirology (n=606, 24.1%), general surgery (n=506, 20.1%), and urinary surgery (n=390, 15.5%). For diseases, pneumonia was the most frequently learned, followed by cholecystolithiasis (n=216, 14.1%), benign prostate hyperplasia (n=196, 12.8%), and bladder tumor (n=193, 12.6%). Among 479 students, roughly a third (n=168, 35.1%) scored in the 60 to 80 range, and half of them scored over 80 points (n=238, 49.7%). The app enabled medical students’ learning to become more active and self-motivated, with a variety of formats, and provided real-time feedback through assessments on the platform. The learning effect was satisfactory overall and provided important precedence for establishing scientific models and methods for assessing clinical thinking skills in the future.

**Conclusions:**

The integration of AI and medical education will undoubtedly assist in the restructuring of education processes; promote the evolution of the education ecosystem; and provide new convenient ways for independent learning, interactive communication, and educational resource sharing.

## Introduction

Clinical thinking refers to the ability of doctors to understand, diagnose, and treat diseases through analysis, synthesis, judgment, reasoning, and other thinking activities by using their medical knowledge, skills, and experience [1]. Clinical reasoning skills include a series of strategies such as correlating information, conducting a comprehensive analysis to form one or more diagnostic hypotheses, weighing the risks and benefits of diagnostic tests and treatments, as well as formulating a reasonable diagnosis and treatment plan [2]. This detailed process includes collecting and evaluating clinical information, selecting diagnostic tests, evaluating test results, developing diagnostic hypotheses, and weighing treatment options and risks [3].

Teaching clinical thinking ability is a challenging task even for experienced clinical teachers. As clinical knowledge is the basis of clinical thinking, the cultivation of clinical thinking needs to go through the links of guidance, deliberate practice, and feedback, and it takes a long time to cultivate efficient clinical thinking ability. The level of clinical thinking of residents is directly related to the safety and quality of medical treatment [4]. Therefore, we should constantly improve the understanding of the clinical thinking training and improve the training quality of residents.

Therefore, the cultivation of clinical thinking ability is a necessary condition for the transition from medical students to clinicians and is currently the key point and difficulty among resident training programs. Among the training processes, the most effective method is based on real or virtual case training, combined with feedback and reflection to continuously improve. Previous studies have shown that clinical thinking ability is scarce among physicians, while offline training cannot meet the gap between the present reality and the ideal requirements [5]. Therefore, there is an urgent need for the development of a web-based database to help physicians learn and exercise clinical thinking ability.

Artificial intelligence (AI) is a novel technology that can be utilized to research and develop the theories, methods, technologies, and applications used to simulate, extend, and expand human intelligence. AI belongs to the interdisciplinary field of natural science, social science, and technological science [6]. AI has a history of more than 70 years, and its application in the medical field mainly focuses on five aspects: medical imaging, auxiliary diagnosis, drug research and development, health management, and disease prediction.

There have been several reports and studies on the use of AI in medical teaching and training of clinical thinking ability. For example, Diagnostic Reasoning (DxR) Clinician (DxR Development Group, Inc.) was developed in 1994 by Myers and Dorsey in the United States to train clinical thinking among students [7]. In 2006, the BP network neural algorithm in AI was applied to teach quality monitoring instead of manually teaching daily monitoring [8]. In 2011, Knewton, an adaptive education platform, offered personalized learning services by extracting learning data from students. In 2017, Tsinghua University launched a study system combined with AI and full quantitative reality virtual technology. In this system, patients’ image data can be converted into a holographic three-dimensional anatomical structure of the human body and mapped in the virtual space [9]. In 2020, a competency-based learning and assessment system (CBLAS) for residency training was designed to provide resident physicians with clinical assessments and learning in order to enhance the learning of trainees and reduce the burden of assessments [10]. However, in the era of AI, there are still issues that exist in medical teaching, such as the lack of general education, the lack of classroom interaction, and the lag in curriculum construction [11].

On this basis, the present study introduces an app named “XueYiKu,” which includes consultations, physical examinations, auxiliary examinations, and diagnosis, by incorporating AI and actual complete hospital medical records to build an online-learning platform using human-computer interaction. This application could cultivate students’ ability to solve clinical problems as well as improve teaching quality and efficiency, thereby achieving the sharing of high-quality teaching resources.

## Methods

### Design and Study Population

The “XueYiKu” app was designed as a contactless, self-service, trial-and-error system application based on actual complete hospital medical records and natural language processing technology to build an online-learning platform using human-computer interaction. Training participants included medical undergraduates, medical graduate students, residents, chief residents, and attending doctors. The purpose of the app was to improve clinical thinking ability through independent study accompanied by real-time feedback. The XueYiKu app was launched on the Android platform on May 14, 2019, and on the IOS platform on August 3, 2019. After 27 updates, the last version of the XueYiKu app was updated on January 12, 2022.

### Data Collection

The general database of the XueYiKu app was based on a collection of real medical records and data from general teaching, including a standard clinical question database, virtual medical record database, and student learning record database. Using information extraction technology in natural language processing from real medical records, the XueYiKu app could poststructure the content from real electronic medical records, generate questions, and match answers online through machine learning. In the teaching cases, the automatically extracted answers matched 90% of the original medical records. The structured cases in the electronic medical record system of the hospital were then screened by a teaching team from Peking University Third Hospital, and appropriate medical records were selected and reviewed by the teaching team. Once the teaching case was determined, relevant inspection results were also extracted, including inspections, auxiliary inspections, and ultrasound, imaging, and pathology findings.

### Outcome Measures

Clinical thinking ability involves extremely high requirements for the objectivity and standardization of evaluation. Hence, a multidimensional automatic evaluation system is required. With reference to the residents’ standardized training report, the whole process of student learning was recorded systematically, and clinical thinking was evaluated in multiple dimensions according to the learning situation of the student, including preciseness, logic, systematicness, agility, knowledge expansion, and multidimensional and comprehensive application (Table 1). Based on the scores of students in various dimensions of learning, a radar chart of clinical thinking ability is drawn to assess the compliance of medical students’ clinical thinking ability.

**Table 1.** Seven elements for clinical thinking evaluation in the XueYiKu app.

Elements	Contents
Preciseness	Comprehensive evaluation of diagnosis and differential diagnosis
Logic	The logical sequence evaluation of inquiry, physical examination, auxiliary examination, and diagnosis
Systematicness	Comprehensive evaluation of the completion rate of inquiry, physical examination, auxiliary examination, diagnosis, and differential diagnosis
Agility	Comprehensive evaluation of the time of inquiry, physical examination, auxiliary examination, diagnosis, and differential diagnosis
Knowledge expansion	Accuracy and logical evaluation of diagnosis and differential diagnosis
Multidimensional evaluation indicators	Comprehensive evaluation of diagnosis and treatment programs
Comprehensive application	Comprehensive application ability of basic and clinical knowledge (for evaluation of level 4 individually)

### Evaluation and Feedback

We recruited all students in Peking University Hospital to evaluate the teaching effect of the XueYiKu app, including medical undergraduates, medical graduate students, residents, chief residents, and attending doctors. Both the questionnaire method and interview method were utilized. Web-based surveys, developed by the study team, were conducted upon completion of the case study so that students could fill in and evaluate their learning effect. Through interviews with relevant teachers and experts, the common clinical path extracted using AI technology was determined. Meanwhile, the range of clinical thinking ability evaluation was confirmed according to the requirements of different training stages. The questionnaire method was used to investigate the following two factors: (1) the current status of clinical thinking training regarding the ability of medical students and residents to clarify the problems in clinical thinking training and improve the content of the database; (2) the experience of medical students and residents regarding the use of the case database and self-evaluation of learning effects to complete the effect evaluation of the XueYiKu app.

### Data Processing

**Figure 1.**
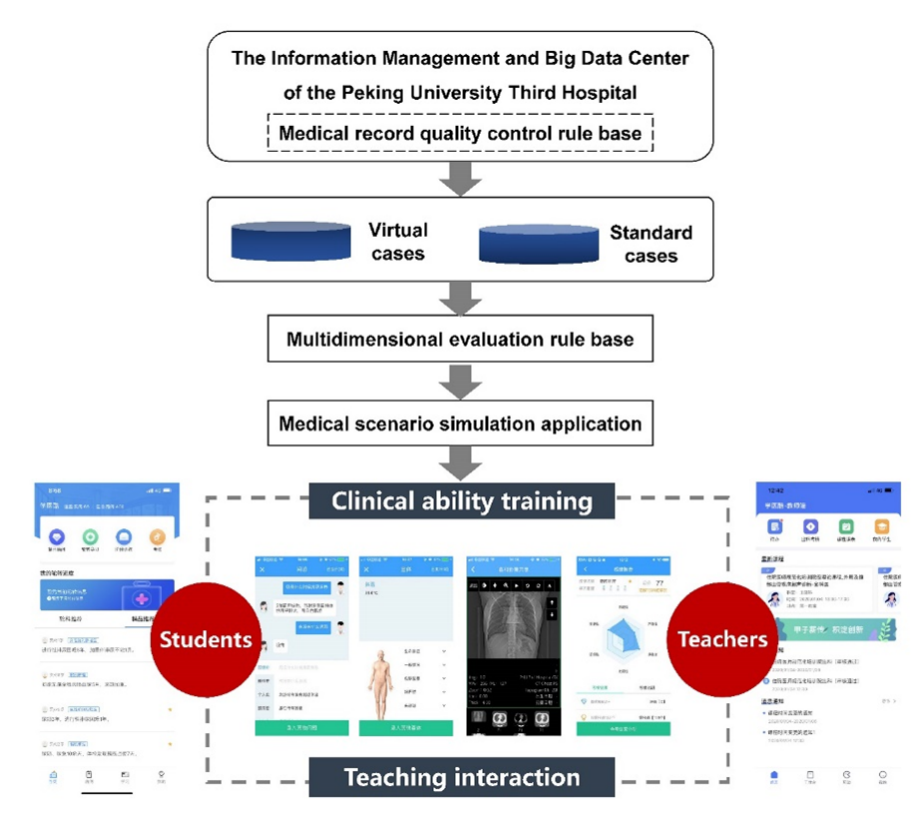
Flow chart of the program design from data collection to interaction between students and teachers in the XueYiKu app.

Details of the virtual case system and AI teaching development based on actual complete hospital medical records and natural language processing technology have been described previously [12]. A flow chart of the program design from data collection to interaction between students and teachers is shown in Figure 1. Case extraction was performed at a hospital’s case data center, and the best-matching cases were extracted through natural language processing, word segmentation, synonym conversion, and sorting. A standard clinical questioning data module, virtual case data module, and student learning difficulty module were established to achieve simulation. Students can view the objective examination and inspection data of actual cases, including details of the consultation and physical examination, and automatically provide their learning response via a multidimensional evaluation system. In order to assess the changes in students’ clinical thinking after using the XueYiKu app, 15 medical graduate students were subjected to two simulation tests before and after learning through the virtual case system. The tests, which included the full-process case examination of cases having the same difficulty level, examined core clinical thinking test points such as consultation, physical examination, and disposal, and generated multidimensional evaluation indicators (rigor, logic, system, agility, and knowledge expansion). Thus, a complete and credible evaluation system was developed.

For statistical analysis, standard descriptive statistics were used to describe the study population: continuous variables were presented as the mean and SD, and categorical variables were presented as percentages.

### Ethical Considerations

This study was reviewed and approved by the Peking University Third Hospital Medical Science Research Ethics Committee (No. IRB00006761-M2022063). Invitations were dispatched to prospective participants via the XueYiKu app, accompanied by a comprehensive informed consent form outlining the nature of the investigation. Participants were clearly notified that the interviews would be audio-recorded solely for analytical purposes and would remain exclusively accessible to the research team. Furthermore, they were assured of their right to withdraw their consent at any juncture during the interview process. All information gathered about the participants was kept private and confidential. All study data were deidentified.

## Results

### Overview

After concealment processing, more than 400 teaching cases covering 65 kinds of diseases were released for students to learn from, and the subjects covered internal medicine, surgery, gynecology and obstetrics, and pediatrics (Table 2). The difficulty in learning cases was divided into 4 levels in ascending order: level 1 provided all score items for students to choose from; except for items from level 1, level 2 provided additional interference items; compared with level 2, level 3 displayed some score items and all interference items to enhance difficulty; for level 4, there was neither a score item nor an interference item, and the system would match the results from the students’ voice or manual input of the questions.

**Table 2.** Categories of subjects and diseases for learning and evaluation in the database of the XueYiKu app.

Subjects	Category	Diseases
Internal Medicine	20	Coronary heart disease, heart failure, arrhythmia, hypertension, pneumonia, chronic airway inflammatory disease, pleural effusion, pulmonary embolism, upper gastrointestinal bleeding, pancreatitis, inflammatory bowel disease, ascites, nephrotic syndrome, acute kidney injury, diabetes, hyperthyroidism, leukemia, anemia, systemic lupus erythematosus, rheumatoid arthritis
Surgery	20	Inguinal hernia, appendicitis, gallbladder stones, bile duct stones, stomach cancer, colon cancer, rectal cancer, liver cancer, pancreatic cancer, lung cancer, pneumothorax, pituitary tumor, coronary heart disease, benign prostatic hyperplasia, bladder tumor, acute respiratory distress syndrome, cervical myelopathy disease, lumbar disc herniation, knee osteoarthritis, ankle fracture
Gynecology and obstetrics	15	Ectopic pregnancy, uterine fibroids, ovarian tumors, dysfunctional uterine bleeding, endometrial cancer, cervical cancer, ovarian cancer, hypertension during pregnancy (including gestational hypertension, preeclampsia, eclampsia, chronic hypertension, and chronic hypertension combined with preeclampsia), gestational diabetes or diabetes (impaired glucose tolerance) combined with pregnancy, prenatal hemorrhage (including placenta previa and abruption of the placenta)
Pediatrics	10	Neonatal hyperbilirubinemia, neonatal respiratory distress syndrome, neonatal sepsis, neonatal hypoxic-ischemic encephalopathy, pneumonia, diarrhea, nutritional diseases, nervous system disease, Kawasaki disease, and kidney disease

### App Use Status

**Figure 2.**
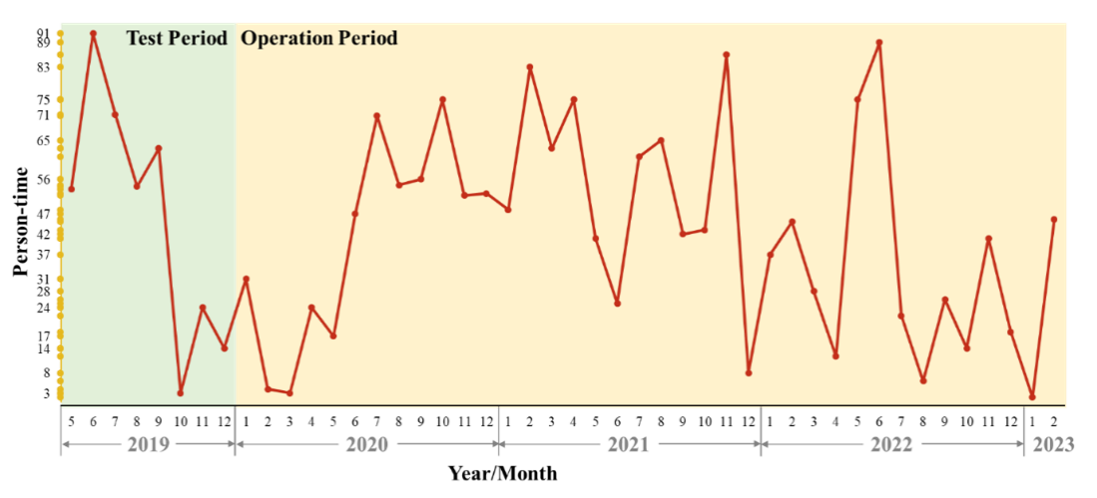
Changes in the number of student users of the XueYiKu app from May 2019 to February 2023.

From the first launch on the Android platform on May 14, 2019, to the last version of the XueYiKu app updated in May 2023, the total number of teacher and student users was 6209 and 1180, respectively. Figure 2 represents the use status of the XueYiKu app during the test period and operation period. In total, there were 3224 person-times in the last 3 years, with an average of 771 person-times per year. From 2019 to 2021, the person-times logged in the application were the highest in 2019, at 1032 (125.4 on average per month), and increased from 646 in 2020 (53.8 on average per month) to 994 in 2021 (76.6 on average per month).

We calculated the frequency at which subjects and related diseases were learned by students, and the top 10 most frequently learned subjects and diseases are listed in Table 3 in descending order. For the second discipline, the most frequently learned subjects were respirology (n=606, 62.2% in internal medicine), general surgery (n=506, 48.8% in surgery), and urinary surgery (n=390, 37.6% in surgery). For diseases, pneumonia was the most frequently learned, with 452 person-times (58.4% in internal medicine), followed by cholecystolithiasis (n=216, 28.4% in surgery), benign prostate hyperplasia (n=196, 25.8% in surgery), and bladder tumor (n=193, 25.4% in surgery).

**Table 3.** The top 10 most frequently learned subjects and diseases in the XueYiKu app.

	Person-time	Percentage (%)
Subjects^a^		
Internal Medicine	975	38.8
Respirology	606	62.2
Cardiology	201	20.6
Gastroenterology	88	9
Endocrinology	80	8.2
Surgery	1036	41.2
General surgery	506	48.8
Urinary surgery	390	37.6
Thoracic surgery	140	13.5
Gynecology and obstetrics	338	13.5
Gynecology	175	51.8
Obstetrics	163	48.2
Pediatrics	163	6.5
Diseases^a^		
Internal Medicine	774	50.4
Pneumonia	452	58.4
Coronary heart disease	99	12.8
Lung cancer	79	10.2
Arrhythmia	76	9.8
Chronic airway inflammation	68	8.8
Surgery	761	49.6
Cholecystolithiasis	216	28.4
Benign prostate hyperplasia	196	25.8
Bladder tumor	193	25.4
Appendicitis	79	10.4
Inguinal hernia	77	10.1

a The top 10 subjects and diseases were listed in descending order due to quantitative limitations.

### Evaluation of the App and its Learning Effects

**Figure 3.**
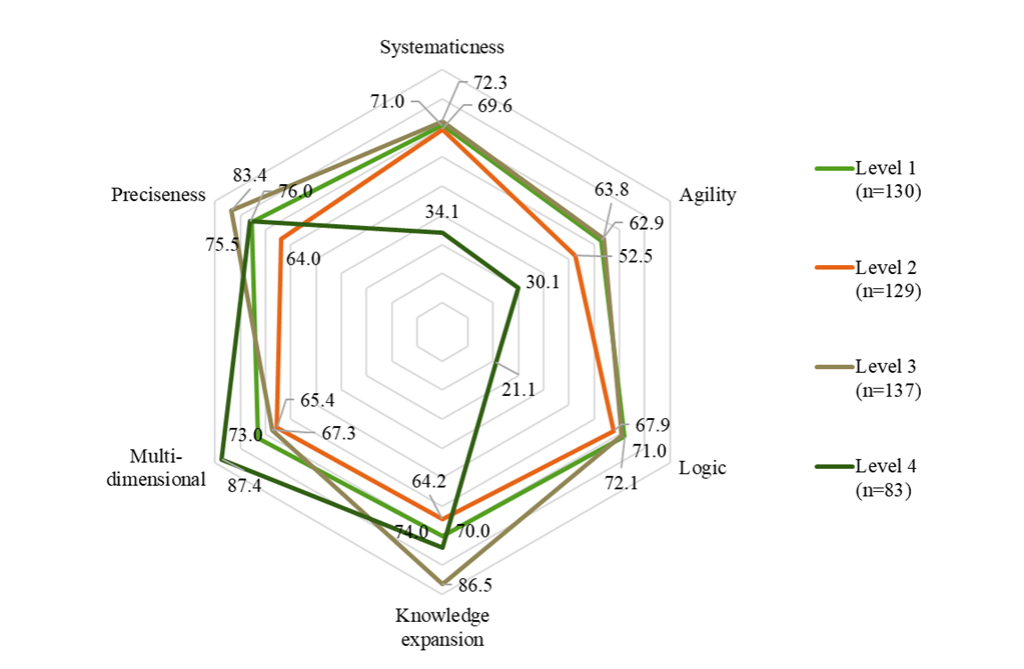
Radar chart with specific scores of clinical thinking evaluation for the XueYiKu app by difficulty in learning cases.

To evaluate the learning effect of the XueYiKu app, the scores of 479 students were analyzed. On the basis of a full score of 100, 15% (n=73) of students scored less than a passing grade of 60 points, roughly a third (n=168, 35.1%) had a score in the 60 to 80 range, and half of them had a score of over 80 points (n=238, 49.7%). A radar chart (Figure 3) with specific scores (Table 4) was presented considering multiple dimensions to evaluate the learning situation of students: students who learned cases with difficulty levels 3‐4 achieved excellent learning outcomes regarding preciseness (83.4 for level 3), multiple dimensions (87.4 for level 4), and knowledge expansion (86.5 for level 3). However, students who learned cases with a difficulty of level 4 did not score as high as the other students in systematicness (34.1 for level 4), agility (30.1 for level 4), and logic (21.1 for level 4). In contrast, students who learned from cases with level 3 difficulty had positive effects in many areas (86.5 in knowledge expansion and 83.4 in preciseness). In fact, there was a high level of satisfaction with knowledge expansion and preciseness in all learners, but it is worth noting that all levels were completely negative about systematicness, agility, and logic.

**Table 4.** Scores of clinical thinking for the XueYiKu app by the difficulty in learning cases and 6 dimensions according to the learning situation of the student.

	Level 1(n=130)	Level 2(n=129)	Level 3(n=137)	Level 4(n=83)
Systematicness	71.0	69.6	72.3	34.1
Agility	62.9	52.5	63.8	30.1
Logic	72.1	67.9	71.0	21.1
Knowledge expansion	70.0	64.2	86.5	74.0
Multidimensional evaluation indicators	73.0	65.4	67.3	87.4
Preciseness	75.5	64.0	83.4	76.0

### Evaluation of the App and Its Learning Effects

**Figure 4.**
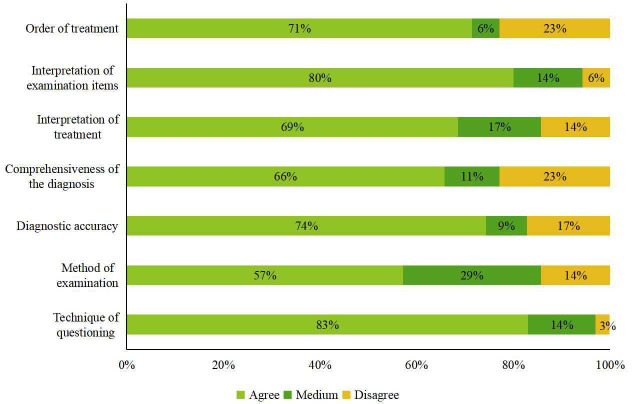
Self-assessment and satisfaction of 70 students towards the learning outcomes for the XueYiKu app.

This section provides information regarding the 70 students’ satisfaction of the XueYiKu app and their self-assessment of its learning effect (Figure 4). Overall, most students were satisfied with the app. The degree of satisfaction kept increasing considering the app function, the personal learning effect, and the case quality. For the self-assessment of learning outcomes, all sections received a rating of more than satisfactory, but the method of examination was rated the least satisfactory. In other sections, the technique of questioning received the highest satisfaction rating, followed by the interpretation of examination items, with both categories receiving more than 80% positive feedback. In contrast, the diagnostic accuracy and the order and interpretation of treatment ranked next in order (~70%), and the other factors were at approximately 60%. Most of the dissatisfaction was rated for the order of treatment and the comprehensiveness of the diagnosis, which were responsible for more than 20% of the negative ratings. Except for the questioning technique, the proportion of negative ratings was more pronounced in all sections. Additionally, for the method of examination, about 30% rated “medium,” which differed greatly from other sections’ ratings.

### Feedback on the App and Its Teaching Effects

According to the feedback, there were some issues associated with the app that are worthy of attention. First, the original login method should be simplified, as well as that for the online case communication and sharing section. Additionally, discussion and comment functions should be added. Regarding data processing, the AI answers could optimize the fuzzy search, and the system could be better connected to the residency system so that students can check and manage the patient’s detailed medical record information. Regarding the case corpus, there is still no section in the app to read images. In addition, not only do the users of the app want to obtain clearer guidance or feedback in future updates, but it would also be beneficial if special departments fulfilled the auxiliary examination and clinical guidelines. Finally, some of the cases included in the app are so simple that they are only suited for undergraduate medical students; it would be beneficial to add cases that are rare, complex, or even life-threatening, and to include the postoperative complications, judgment, treatment, and other information relevant to the case.

## Discussion

### Principal Findings

Clinical thinking training for medical students is very important; however, few design cases rely on IT for self-learning. The current study uses AI and big data platforms to establish a virtual learning platform based on real clinical cases. The XueYiKu app can assist in the development of medical students’ clinical thinking skills by allowing them to perform consultation, physical examination, auxiliary examination, diagnosis, and treatment on virtual patients; the app can also design real cases with different difficulties to meet the needs of residents’ clinical thinking training at different stages. Through the evaluation of students’ experiences with the app, this study concludes that medical students’ learning can become more active and self-motivated, using a variety of formats, with real-time feedback through the platform’s assessment. The learning effect is also satisfactory overall and provides important methods and ideas for establishing scientific models and methods for assessing clinical thinking skills in the future.

### Comparison to Prior Work

Analysis of the data shows that the number of case studies was positively correlated with the number of residents in the specialty. The top 4 diseases most frequently learned included pneumonia, cholecystolithiasis, benign prostate hyperplasia, and bladder tumor, all of which are common, require adequate diagnosis and differential diagnosis, and meet the basic goal of clinical thinking training at the residency stage. The platform contains 65 common diseases and 400 study cases covering internal medicine, surgery, obstetrics and gynecology, and pediatrics. The design is reasonable and can meet the basic learning needs of residents in various specialties. In the 1990s, DxR Clinician was developed by DxR Development Group, Inc. [7] in the United States, and it was later introduced and translated into Chinese by the China Union Medical University Press in 2009 [13]. However, the use of this software in China is still in its infancy, with few relevant literature reports. A previous study showed that the cases included in the DxR software were virtual cases and were limited by the purchase [7]. Students needed to complete the study on the computer, and the average time for students to become familiar with the software was as long as 2 hours. In contrast, the types of diseases and the number of cases covered in the XueYiKu app, as well as the ease of use, are advantages. In addition, the framework of this platform is reasonable, as new case types of diseases can be added according to students’ needs, and the user interface is simple and fast. Therefore, this app is more suitable for residents’ clinical thinking training in the global internet and postepidemic era.

There is a decline in the training and formation of clinical thinking in residents. Based on this, the real case base used in this app was designed to present 4 levels of difficulty for the same case. Evaluation and feedback in the process of clinical thinking training formation and refinement are also important. As a result, students were evaluated in multiple dimensions according to the learning situation. Analysis of the results showed that the learning process meets the clinical diagnosis and treatment reality. For first- to second-year residents, level 2‐3 difficulty was the most appropriate, as illustrated by their better performance in knowledge expansion and logic, among other factors. The clinical learning process is in line with clinical practice and meets the learning needs of residents. Specific to the learning process at different levels of difficulty, the primary difficulty allowed for a complete diagnosis based on the completed clinical information, and as the difficulty level increased, there was an advancement of the learning model; the results of the 4 levels of difficulty were in line with the development of the resident’s clinical thinking, realizing the intended purpose of the app. One similar app currently in use is Brianly, a medical tutoring and social networking platform that provides personalized medical learning tutoring through AI algorithms [14]. In Tsinghua University, “artificial intelligence + virtual reality” within Brianly is used for clinical training, which allows direct viewing of the anatomical details of a patient’s real body structure in a virtual space, as well as virtual surgery. However, there is currently no learning software for medical students that is designed to match the growth and learning process, and that provides a difficulty gradient based on the clinical thinking ability. In this context, our current app will continue to develop, improve, and provide feedback related to the 4 levels of difficulty to be better used for the clinical thinking training of senior residents.

Based on current usage, the highest number of uses per capita was usually found during the admission and start of residency, that is, from September to February each year. The survey results show that students had a high level of satisfaction with the consultation aspect of the app. The case study on the app allows for both a generic history-taking process and a structured questioning for a particular disease, which is inextricably linked to the teaching case base from clinical consultations of real cases. However, there are some differences between the examination and the actual situation, and the contents of the specialist examination and the physical examination for a particular disease should be further optimized to be more concise and focused.

### Strengths and Limitations

One of the greatest advantages of our app is the inclusion of real cases, which could provide a new model of clinical thinking training by building a system to comprehensively assess the “clinical competence” of residents at different stages. For students, the strongest advantage is that they can customize clinical competency training according to their needs without the limitations of time and space. For app designers, the XueYiKu app is inherently user-friendly and sustainable, which allows the addition of cases in bulk based on the existing framework.

In addition to the benefits of the app, there are several limitations that could be further improved. First, current cases and diseases could be further expanded to cover more subjects, which is the next step of development. Second, in terms of use, students currently use the app independently, while the frequency of use is mainly based on students’ interests combined with required course study and clinical rotation. Third, current evaluation results are almost subjective evaluations by humans. Thus, the evaluation results may be biased.

### Conclusion

The emergence of virtual network learning systems such as the XueYiKu app can not only rapidly cultivate students’ ability to solve clinical problems but also save teaching costs, improve teaching quality and efficiency, and achieve the sharing of high-quality teaching resources. In addition to enriching the content of clinical teaching, students with diverse experience and innovative thinking are trained, which promotes the basic clinical knowledge of medical students and effectively cultivates their clinical thinking ability.

## Abbreviations

AI: artificial intelligence
CBLAS: competency-based learning and assessment system
DxR: diagnostic reasoning

## References

[1] Richards JB, Hayes MM, Schwartzstein RM (2020). Teaching clinical reasoning and critical thinking: from cognitive theory to practical application. Chest.

[2] Reinus JF (2022). Patient-data management and clinical reasoning: neglected essential skills. Am J Med.

[3] Karunaratne D, Sibbald M, Chandratilake M (2024). Understanding cultural dynamics shaping clinical reasoning skills: A dialogical exploration. Med Educ.

[4] Locke R, Mason A, Coles C, Lusznat RM, Masding MG (2020). The development of clinical thinking in trainee physicians: the educator perspective. BMC Med Educ.

[5] Lee DS, Abdullah KL, Subramanian P, Bachmann RT, Ong SL (2017). An integrated review of the correlation between critical thinking ability and clinical decision-making in nursing. J Clin Nurs.

[6] Carin L (2020). On artificial intelligence and deep learning within medical education. Acad Med.

[7] Myers JH, Dorsey JK (1994). Using diagnostic reasoning (DxR) to teach and evaluate clinical reasoning skills. Acad Med.

[8] Xu X, Liu F (2021). Optimization of online education and teaching evaluation system based on GA-BP neural network. Comput Intell Neurosci.

[9] Nagi F, Salih R, Alzubaidi M (2023). Applications of artificial intelligence (AI) in medical education: a scoping review. Stud Health Technol Inform.

[10] Hsiao CT, Chou FC, Hsieh CC, Chang LC, Hsu CM (2020). Developing a competency-based learning and assessment system for residency training: analysis study of user requirements and acceptance. J Med Internet Res.

[11] Webster CS (2021). Artificial intelligence and the adoption of new technology in medical education. Med Educ.

[12] Wang M, Sun Z, Jia M (2022). Intelligent virtual case learning system based on real medical records and natural language processing. BMC Med Inform Decis Mak.

[13] Meng Q, Zhaoxia T (2012). Promoting effect of information products on case-based clinical teaching model. J Med Inform.

[14] Han ER, Yeo S, Kim MJ, Lee YH, Park KH, Roh H (2019). Medical education trends for future physicians in the era of advanced technology and artificial intelligence: an integrative review. BMC Med Educ.

